# Individual Freedom in the Initial Response to Covid-19

**DOI:** 10.3389/fpubh.2022.765016

**Published:** 2022-06-03

**Authors:** Salvador Macip, Oriol Yuguero

**Affiliations:** ^1^Mechanisms of Cancer and Aging Laboratory, Department of Molecular and Cell Biology, University of Leicester, Leicester, United Kingdom; ^2^Food Lab, Faculty of Health Sciences, Universitat Oberta de Catalunya, Barcelona, Spain; ^3^Emergency Research Group, Biomedical Research Institute of Lleida (IRBLLEIDA), Lleida, Spain

**Keywords:** COVID-19, ethics, pandethics, syndemic, individual freedom

## Abstract

The COVID-19 pandemic has been a phenomenal challenge to global health care and will continue to be so in the upcoming months. Beyond its medical toll, COVID-19 has also exacerbated pre-existing social issues and created new inequalities. This has generated a series of ethical problems that will need to be carefully analyzed to avoid repeating similar mistakes in the context of other crises. Among those, we discuss here the bioethical implications of preserving individual freedom in the context of the early response to a pandemic and propose a global approach to the issue that could be applied in future health challenges.

## Introduction

The COVID-19 crisis that started at the end of 2019 and spread over the world in early 2020 highlighted different ethical concerns in a wide range of situations. In fact, the term pandethics was coined precisely to describe and study the ethical issues associated with a pandemic (1), which can be numerous but often tend to be overlooked. Factoring ethical parameters when making global health decisions in a pandemic may be difficult, since priorities rightly tend to lie elsewhere (saving lives, chiefly). This is the reason why it is particularly important to discuss and solve these ethical issues ahead of the need for a timely response. Although it may be late for COVID-19, we can still learn from the mistakes made in the past years and apply the conclusions of our analyses in future crises.

A syndemic (a portmanteau of “synergistic epidemics”) is the simultaneous occurrence of two or more epidemics, a word originally used to describe the concurrence of infectious diseases (2) such as hookworm, malaria and AIDS (3). This definition was later stretched to include also non-communicable diseases, such as stress and obesity (4). It has been proposed that the COVID-19 pandemic should, in fact, be considered a syndemic due to the co-prevalence of other health conditions in economically disadvantaged social groups (5) or the fact that elderly populations [ageing itself could be considered a disease (6)] and those with underlying conditions are most at risk of suffering severe symptoms (7).

In addition, we believe that mental health should be considered a fundamental element to support the idea that COVID-19 needs to be considered a syndemic. It has been shown that the different lockdowns put in place in many countries over the pandemic caused an increase in intrafamily violence, as well as an increase in cases of suicide and imbalances in mental health disorders (8). This affected not only regular citizens, but also health professionals, who were forced to work extra hours in a health system that was overwhelmed with little recognition for their efforts. It is fair to assume that many of the long-term consequences of COVID-19 will not only be physical, but also mental.

It would be important to further expand the range of the term to include other burdens implicit in any global health crisis, which should be considered a simultaneous pandemic on their own. Following this view, the COVID-19 syndemic would involve not only the biological effects of the virus, added to other already present and independent health problems, but also a secondary multi-headed social pandemic that needs to be addressed simultaneously. This would include economic, cultural and ethical branches, among others. Out of the many components of its ethical dimension, some of which we have summarized in Table 1, we believe individual freedom needs to be given special consideration.

**Table 1:** Examples of principal pandethic challenges in the COVID-19 syndemic.

Age-dependent prioritization of patients in overwhelmed intensive care units. Lack of adequate support and care for non-covid patients. Lack of resources to non-hospital assistance for COVID-19 and non-COVID-19 patients. Shortage of funds in non-covid research aeras. Lack of access to other essential biomedical research under lockdown restrictions Unequal distribution of cases, with higher burden in lowest socioeconomic backgrounds. Unequal access to diagnosis, treatments and vaccines. Lack of a global response planned with input from all stakeholders. Administering untested drugs to severely affected patients. Clinical trial designs without all ethical safeguards. Isolation of the elderly population. Loneliness at the end of life of patients with COVID-19. Freedom of speech vs. spreading disinformation. Governmental control of data and communications. General loss of individual freedom.

## The Paradox of Individual Freedom

Controlling the COVID-19 pandemic proved to be particularly difficult in the initial stages, before vaccines were available. Scientific evidence shows that countries with greater capacity to restrict mobility and some essential freedoms were initially more successful at thwarting the progress of the virus (9). In many Western countries, the establishment of curfews, lockdowns and various forms of states of emergency/alarm were perceived as an attack on the citizens' fundamental rights. These territories, where restriction measures were, as a consequence, laxer than in other countries, did not achieve a good management of rate of contagion in the first months of the pandemic (10). In Southeast Asia, for instance, various surveillance mechanisms made it possible to trace the movements and activity of millions of people, thus being visible to government institutions 24 hours per day (11). This likely played an important role in dampening the first wave of COVID-19 in these territories, while Europe and the Americas, reluctant to implement such invasive measures, struggled.

Societies with higher scores in the EIU democracy index (12), which usually achieved their historical liberties many decades ago, seem to be hesitant to relinquish too much of their personal sovereignty to control a health crisis, even if the outcome is an increase in the overall survival of the population. This poses an ethical dilemma of individual freedom superseding the social responsibility of minimizing the impact of a transmissible disease.

However, it needs to be considered who has more freedom in the end—those who can go wherever they want while being watched or those who can only go out for essential purposes but without anyone controlling their movements. The paradox is that, in the long run, temporary loss of freedom may grant greater personal liberty than an extreme preservation of individual rights, thus resulting in a higher societal benefit. The ideal situation would be to strike a balance between control and freedom, in which an acceptable degree of personal sacrifice is socially agreed. This can only be achieved after careful discussion and negotiation with all the stakeholders, and should thus be done before the emergence of a health crisis. In the case of COVID-19, it was seen that the management of individual freedom presented a new point of imbalance for western democracies (13), many of which arrived at the pandemic already struggling due to the ongoing social movements claiming more transparency.

## How to Best Surrender Privacy

Temporarily relinquishing personal rights can only be tolerated when the proper safety measures are in place. In western societies, the custodian of personal information is often mistrusted, since privacy is seen as one of the most valued treasures. However, with the adequate supervision and full warranties to avoid abuses of power by the government, a temporal restriction of freedom in the face of a new pandemic would have the expected benefit of reducing mortality, as discussed above, and would also lack unwanted side effects in the form of unacceptable losses of liberty.

It can be debated whether an absolute guarantee is possible in this context. Indeed, the custody of personal information contained in Big Data has always raised ethical dilemmas about surveillance and access (14). In recent years, large collaborative studies have been performed, in which substantial amounts of patient and citizen data have been shared and used by the many parties involved. As a result, data protection has been increased to prevent large companies and others with commercial interests from accessing the information that volunteers give freely when participating in scientific research. Similarly, in the event of a global health crisis, the first step should be for governments to establish independent agencies and control mechanisms that could ensure, as much as possible, the confidentiality of the data obtained from citizens. Periodic external audits and controls could help to avoid the misuse of this sensitive information, protect the population, enhance trust and thus foster the compliance of the largest percentage possible.

## The Ethical Implications of Information Flow in a Pandemic

An essential component of personal freedom is the access to reliable facts to inform any decision. Without this, informed consent to safely surrender individual freedom is impossible. This is particularly challenging in the current era of information overload due to the widespread use of social media. During this pandemic, there have been many examples of misleading propaganda driving personal choices toward unsafe options. For instance, while COVID-19 vaccination was becoming a reality in most developed countries, valid information was mixed with opinions that casted serious doubts on the safety of the campaign without presenting any scientific proof to substantiate the claims. Rare adverse effects were maximized to spread distrust on vaccines and their development, leading to many citizens making decisions detrimental for both their wellbeing and public health.

Faced with the worst pandemic in decades, people reacted to public statements with suspicion, and often preferred to listen to fringe sources that doubted the official information. This is a worldwide trend that needs to be factored in when designing official communication strategies in a pandemic, which are key to protect individual liberties. The fact that questioning the system attracts wide audiences than following official regulations can be particularly dangerous when social responsibility is at stake. If the general population lacks the basic scientific knowledge to be able to identify reliable sources in a time of crisis, personal freedom will be affected and global health will suffer. Thus, scientists and policy makers need to analyse what responsibility they may have in the current mistrust of the official sources of information and how can this be addressed in time for the next global health challenge.

There is a deontological responsibility of the scientific community to dispel society's fears, but there is also an ethical duty to take a step aside to avoid personalisms that blur an objective and rational message. It is clear that individual efforts will not be sufficient to compensate for the bad management of scientific knowledge and the widespread persistence of disinformation. It would be preferable that the experts pool all their knowledge to create an objective and rigorous communication system, made of a wide range of scientists of all fields, including social disciplines, that could independently advise governments. Scientific autonomy from political and economic influence is essential to find solutions, regain the trust of the population, and prevent the mistakes that plagued the beginning of the pandemic, as recognized by various experts (15). To achieve this, scientists have the ethical responsibility to put personal quarrels and political inclinations aside in benefit of a greater good and a clearer communication.

## The Responsibility of Leaders

Individual freedom is also restricted when governments fail to protect the rights of the citizens by taking ill-advised decisions. The pandemic has affected countries governed by all sorts of political systems. We have seen how successful models of management that succeeded in the first waves collapsed under the pressure of successive ones. There has been the constant dilemma of choosing between protecting the economy or prioritizing health, when this should be seen as a false conundrum—economy is essential to guarantee health and that a sick society is unable to drive a strong economy. This syndemic has shown that, in many cases, the base of a country's economic structure was weak and fragile. It has also highlighted social inequalities that were already known but widely ignored. The risk now is to not take this opportunity to solve these long-standing issues, from the dire conditions of seasonal workers in many countries to the lack of attention given to elderly care.

Present and future leaders have the responsibility to strengthen the social tissue in all these areas so it can better withstand situations of extreme stress, such as those seen in a pandemic. COVID-19 has often exposed a lack of humility of politicians when assuming the inherent difficulties of the situation and the challenges of controlling it, recognizing mistakes, and, in the face of uncertainty, seeking advice from those who know more. Above all, society needs to find an alternative to its fascination with overconfident leaders that fail to admit their shortcomings.

In the face of these situations, we need to ask what can be done. It is essential to understand that, from now on, public health must be considered in a global way—as planetary health. The COVID-19 crises taught us that we cannot control a pandemic until all the countries achieve a similar level of containment.

## The Need of a Global Response

COVID-19 has shown that, despite the huge advances in pandemic management that vaccination may entail at a local level, global immunization needs to be achieved to ensure that the crises is finished. This is another factor that limits personal freedom due to the unequal opportunities in different parts of the world. While most high-income countries now show very encouraging vaccination rates, we view with concern the uncontrolled situation of the pandemic in other areas. While the same rate of vaccination and coverage are not reached globally, inequalities will prevail, and the emergence of new variants that could escape the protection that vaccines provide will always be a risk. This underscores the importance of acting co-ordinately, and the role that international organizations such as WHO (16) should play in the management of a pandemic. In addition, it would also be important to reconsider the global role of WHO, which has failed on several occasions to take the lead in controlling the pandemic response and currently does not have the worldwide support and recognition it should have.

As hinted above, COVID-19 has also shown the importance of promoting independent scientific institutions and boards. Non-governmental organizations that report transparently and make proposals based on epidemiological data and not on the basis of non-scientific criteria, as seems to have happened many times and often generate anti-system sentiments. This is also a way of prop up democracy and individual freedom through the health system.

## Concluding Remarks

Global crises pose phenomenal challenges, but also introduce the opportunity to solve issues that tend to be neglected in normal situations. We need to take advantage of this situation and analyse the factors that have contributed to the most deficient responses to the pandemic (weak leadership, lack of scientific culture, lack of coordination, lack of self-criticism, poor preparation, poor communication, long reaction times, individualism, etcetera) and propose feasible solutions to be implemented before the next crisis. Pandethics needs to feature prominently in these discussions, as a way to highlight the weakest points, with special focus on how to preserve personal freedom while protecting the greater good. Failure to do so will likely lead to avoidable suffering the next time the system is stressed. We believe is essential to start these discussions as soon as possible, since finding a solution to these issues will take careful analysis and long consultations.

## Author Contributions

All authors listed have made a substantial, direct, and intellectual contribution to the work and approved it for publication.

## Funding

This work was supported by the M.C. Andreu Memorial Fund.

## Conflict of Interest

The authors declare that the research was conducted in the absence of any commercial or financial relationships that could be construed as a potential conflict of interest.

## Publisher's Note

All claims expressed in this article are solely those of the authors and do not necessarily represent those of their affiliated organizations, or those of the publisher, the editors and the reviewers. Any product that may be evaluated in this article, or claim that may be made by its manufacturer, is not guaranteed or endorsed by the publisher.
